# Supplementary material for: Inferring gender from first names: Comparing the accuracy of Genderize, Gender API, and the gender R package on authors of diverse nationality
Source: PLOS Digit Health. 2024 Oct 29;3(10):e0000456. doi: 10.1371/journal.pdig.0000456 (PMC11521266; doi:10.1371/journal.pdig.0000456)
Supplement: S1 Fig — The HemOnc KB is a growing resource, and 4,360 trialists had not had their genders evaluated at the time of this study. Of the 32,968 trialists included in the study, 24,930 were affiliated with sites in a single country. (DOCX) [file pdig.0000456.s001.docx]

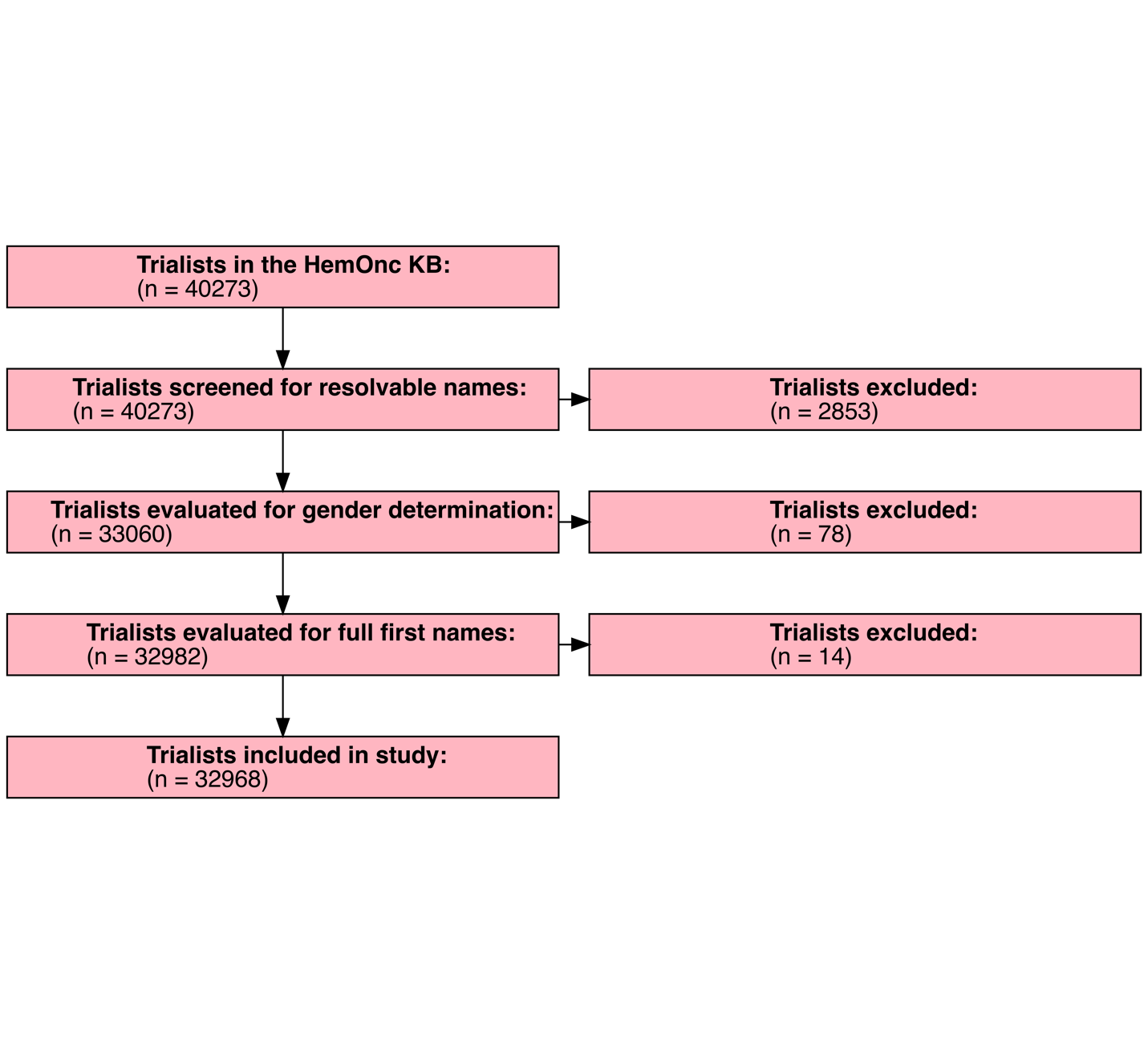


**Supp. Fig 1: Flowchart depicting trialist inclusions from HemOnc Knowledgebase (KB).** The HemOnc KB is a growing resource, and 4,360 trialists had not had their genders evaluated at the time of this study. Of the 32,968 trialists included in the study, 24,930 were affiliated with sites in a single country.
