# Supplementary material for: Inferring gender from first names: Comparing the accuracy of Genderize, Gender API, and the gender R package on authors of diverse nationality
Source: PLOS Digit Health. 2024 Oct 29;3(10):e0000456. doi: 10.1371/journal.pdig.0000456 (PMC11521266; doi:10.1371/journal.pdig.0000456)
Supplement: S7 Table — (DOCX) [file pdig.0000456.s008.docx]

**Supplemental Table 7.** 100 Most Common Trialist Name-Country Combinations

| **Name** | **Country** | **Men with Name** | **Women with Name** | **Genderize Prediction** | **Gender API Prediction** |
| --- | --- | --- | --- | --- | --- |
| david | USA | 201 | 0 | male | male |
| michael | USA | 196 | 0 | male | male |
| john | USA | 175 | 0 | male | male |
| robert | USA | 163 | 0 | male | male |
| james | USA | 133 | 0 | male | male |
| william | USA | 126 | 0 | male | male |
| richard | USA | 101 | 0 | male | male |
| thomas | USA | 94 | 0 | male | male |
| mark | USA | 92 | 0 | male | male |
| daniel | USA | 83 | 0 | male | male |
| jeffrey | USA | 79 | 0 | male | male |
| paul | USA | 78 | 0 | male | male |
| jennifer | USA | 0 | 72 | female | female |
| peter | USA | 67 | 0 | male | male |
| joseph | USA | 64 | 0 | male | male |
| steven | USA | 63 | 0 | male | male |
| charles | USA | 62 | 0 | male | male |
| michael | Germany | 61 | 0 | male | male |
| susan | USA | 0 | 61 | female | female |
| christopher | USA | 60 | 0 | male | male |
| david | United Kingdom | 58 | 0 | male | male |
| stephen | USA | 58 | 0 | male | male |
| andrew | USA | 56 | 0 | male | male |
| martin | Germany | 54 | 0 | male | male |
| maria | Italy | 0 | 53 | female | female |
| maria | Spain | 1 | 51 | female | female |
| brian | USA | 51 | 0 | male | male |
| jose | Spain | 51 | 0 | male | male |
| scott | USA | 50 | 0 | male | male |
| elizabeth | USA | 0 | 49 | female | female |
| eric | USA | 48 | 0 | male | male |
| matthew | USA | 48 | 0 | male | male |
| francesco | Italy | 47 | 0 | male | male |
| giuseppe | Italy | 44 | 0 | male | male |
| philippe | France | 44 | 0 | male | male |
| christian | Germany | 42 | 0 | male | male |
| edward | USA | 42 | 0 | male | male |
| gary | USA | 41 | 0 | male | male |
| kenneth | USA | 41 | 0 | male | male |
| john | United Kingdom | 40 | 0 | male | male |
| maria | USA | 0 | 40 | female | female |
| takashi | Japan | 39 | 0 | male | male |
| thomas | Germany | 39 | 0 | male | male |
| jonathan | USA | 38 | 0 | male | male |
| mary | USA | 0 | 38 | female | female |
| timothy | USA | 38 | 0 | male | male |
| peter | Germany | 37 | 0 | male | male |
| andreas | Germany | 36 | 0 | male | male |
| karen | USA | 0 | 36 | female | female |
| george | USA | 35 | 0 | male | male |
| **Name** | **Country** | **Men with Name** | **Women with Name** | **Genderize Prediction** | **Gender API Prediction** |
| hiroshi | Japan | 35 | 0 | male | male |
| anthony | USA | 34 | 0 | male | male |
| bruce | USA | 34 | 0 | male | male |
| patrick | USA | 34 | 0 | male | male |
| philip | USA | 34 | 0 | male | male |
| pierre | France | 34 | 0 | male | male |
| andrew | United Kingdom | 33 | 0 | male | male |
| lisa | USA | 0 | 33 | female | female |
| nancy | USA | 0 | 33 | female | female |
| giovanni | Italy | 31 | 0 | male | male |
| katherine | USA | 0 | 31 | female | female |
| antonio | Italy | 30 | 0 | male | male |
| patricia | USA | 0 | 30 | female | female |
| roberto | Italy | 30 | 0 | male | male |
| gregory | USA | 29 | 0 | male | male |
| kevin | USA | 29 | 0 | male | male |
| olivier | France | 29 | 0 | male | male |
| richard | United Kingdom | 29 | 0 | male | male |
| sarah | USA | 0 | 29 | female | female |
| alan | USA | 28 | 0 | male | male |
| paolo | Italy | 28 | 0 | male | male |
| peter | United Kingdom | 28 | 0 | male | male |
| amy | USA | 0 | 27 | female | female |
| donald | USA | 27 | 0 | male | male |
| douglas | USA | 27 | 0 | male | male |
| lawrence | USA | 27 | 0 | male | male |
| martin | USA | 27 | 0 | male | male |
| anne | USA | 0 | 26 | female | female |
| david | Canada | 26 | 0 | male | male |
| hans | Germany | 26 | 0 | male | male |
| julie | USA | 0 | 26 | female | female |
| marco | Italy | 26 | 0 | male | male |
| michael | United Kingdom | 26 | 0 | male | male |
| stefan | Germany | 26 | 0 | male | male |
| andrea | Italy | 25 | 0 | female | female |
| barbara | USA | 0 | 25 | female | female |
| deborah | USA | 0 | 25 | female | female |
| laura | USA | 0 | 25 | female | female |
| michael | Australia | 25 | 0 | male | male |
| alessandro | Italy | 24 | 0 | male | male |
| alexander | USA | 24 | 0 | male | male |
| christoph | Germany | 24 | 0 | male | male |
| eric | France | 24 | 0 | male | male |
| francesca | Italy | 0 | 24 | female | female |
| frank | USA | 24 | 0 | male | male |
| howard | USA | 24 | 0 | male | male |
| jason | USA | 24 | 0 | male | male |
| rebecca | USA | 0 | 24 | female | female |
| ronald | USA | 24 | 0 | male | male |
| matthias | Germany | 23 | 0 | male | male |
