# Supplementary material for: Inferring gender from first names: Comparing the accuracy of Genderize, Gender API, and the gender R package on authors of diverse nationality
Source: PLOS Digit Health. 2024 Oct 29;3(10):e0000456. doi: 10.1371/journal.pdig.0000456 (PMC11521266; doi:10.1371/journal.pdig.0000456)
Supplement: S8 Table — (DOCX) [file pdig.0000456.s009.docx]

**Supplementary Table 8: Precision, Recall, and F1 Scores**

|  | **Man set as Positive ^a^** | | | **Woman Set as Positive ^a^** | | | **Global Statistics ^b^** | | |
| --- | --- | --- | --- | --- | --- | --- | --- | --- | --- |
| **Method** | **Precision** | **Recall** | **F1** | **Precision** | **Recall** | **F1** | **Precision** | **Recall** | **F1** |
| Gender R Package IPUMS | 0.953 | 0.972 | 0.962 | 0.946 | 0.912 | 0.928 | 0.951 | 0.832 | 0.887 |
| Gender R Package SSA | 0.994 | 0.974 | 0.984 | 0.954 | 0.99 | 0.972 | 0.98 | 0.873 | 0.923 |
| Gender Api No Countries in API Call | 0.976 | 0.982 | 0.979 | 0.965 | 0.955 | 0.96 | 0.972 | 0.988 | 0.98 |
| Gender Api No Countries in API Call; Only Trialists with a Recorded Country ^c^ | 0.974 | 0.98 | 0.977 | 0.963 | 0.951 | 0.957 | 0.97 | 0.987 | 0.978 |
| Gender Api with Countries in API Call | 0.979 | 0.979 | 0.979 | 0.96 | 0.962 | 0.961 | 0.973 | 0.986 | 0.979 |
| Genderize No Countries in API Call | 0.983 | 0.981 | 0.982 | 0.965 | 0.968 | 0.966 | 0.977 | 0.989 | 0.983 |
| Genderize No Countries in API Call; Only Trialists with a Recorded Country ^c^ | 0.981 | 0.98 | 0.98 | 0.962 | 0.964 | 0.963 | 0.974 | 0.988 | 0.981 |
| Genderize with Countries in API Call | 0.985 | 0.982 | 0.984 | 0.967 | 0.971 | 0.969 | 0.979 | 0.974 | 0.976 |

^a^ When calculating Precision, Recall, and F1 scores, one gender was set as the positive prediction, and the other gender was considered the negative prediction. Names that yielded no gender predictions were excluded.

^b^ Men’s names correctly gendered and women’s names correctly gendered were classified as true positives. Names that yielded no gender predictions were classified as false negatives.

^c^ Only names that were labeled with an affiliated country were included. Countries were not included in the API call.
