# Supplementary material for: Inferring gender from first names: Comparing the accuracy of Genderize, Gender API, and the gender R package on authors of diverse nationality
Source: PLOS Digit Health. 2024 Oct 29;3(10):e0000456. doi: 10.1371/journal.pdig.0000456 (PMC11521266; doi:10.1371/journal.pdig.0000456)
Supplement: S9 Table — (DOCX) [file pdig.0000456.s010.docx]

**Supplemental Table 9.** Gender Prediction Accuracy For all Countries with at Least 20 Trialists When Countries are Not Included in the API Call

|  |  | **Genderize** | | | **Gender API** | | |
| --- | --- | --- | --- | --- | --- | --- | --- |
| **Country** | **Total, n** | **Correct, n (%)** | **Incorrect, n (%)** | **No Predictions, n (%)** | **Correct, n (%)** | **Incorrect, n (%)** | **No Predictions, n (%)** |
| Total | 24929 | 23993/24929 (96.2%) | 636/24929 (2.6%) | 300/24929 (1.2%) | 23872/24929 (95.8%) | 735/24929 (2.9%) | 322/24929 (1.3%) |
| USA | 9485 | 9161/9485 (96.6%) | 244/9485 (2.6%) | 80/9485 (0.8%) | 9095/9485 (95.9%) | 301/9485 (3.2%) | 89/9485 (0.9%) |
| France | 1868 | 1816/1868 (97.2%) | 36/1868 (1.9%) | 16/1868 (0.9%) | 1805/1868 (96.6%) | 47/1868 (2.5%) | 16/1868 (0.9%) |
| Germany | 1853 | 1832/1853 (98.9%) | 15/1853 (0.8%) | 6/1853 (0.3%) | 1827/1853 (98.6%) | 17/1853 (0.9%) | 9/1853 (0.5%) |
| Italy | 1754 | 1702/1754 (97%) | 49/1754 (2.8%) | 3/1754 (0.2%) | 1712/1754 (97.6%) | 39/1754 (2.2%) | 3/1754 (0.2%) |
| United Kingdom | 1713 | 1679/1713 (98%) | 25/1713 (1.5%) | 9/1713 (0.5%) | 1675/1713 (97.8%) | 31/1713 (1.8%) | 7/1713 (0.4%) |
| Japan | 1169 | 1148/1169 (98.2%) | 9/1169 (0.8%) | 12/1169 (1%) | 1129/1169 (96.6%) | 24/1169 (2.1%) | 16/1169 (1.4%) |
| Canada | 773 | 748/773 (96.8%) | 16/773 (2.1%) | 9/773 (1.2%) | 755/773 (97.7%) | 11/773 (1.4%) | 7/773 (0.9%) |
| Spain | 726 | 710/726 (97.8%) | 16/726 (2.2%) | 0/726 (0%) | 713/726 (98.2%) | 13/726 (1.8%) | 0/726 (0%) |
| Netherlands | 663 | 647/663 (97.6%) | 11/663 (1.7%) | 5/663 (0.8%) | 640/663 (96.5%) | 16/663 (2.4%) | 7/663 (1.1%) |
| China | 631 | 513/631 (81.3%) | 96/631 (15.2%) | 22/631 (3.5%) | 485/631 (76.9%) | 110/631 (17.4%) | 36/631 (5.7%) |
| Switzerland | 484 | 471/484 (97.3%) | 7/484 (1.4%) | 6/484 (1.2%) | 469/484 (96.9%) | 9/484 (1.9%) | 6/484 (1.2%) |
| Australia | 475 | 463/475 (97.5%) | 9/475 (1.9%) | 3/475 (0.6%) | 459/475 (96.6%) | 11/475 (2.3%) | 5/475 (1.1%) |
| South Korea | 324 | 257/324 (79.3%) | 25/324 (7.7%) | 42/324 (13%) | 258/324 (79.6%) | 22/324 (6.8%) | 44/324 (13.6%) |
| Belgium | 313 | 298/313 (95.2%) | 14/313 (4.5%) | 1/313 (0.3%) | 295/313 (94.2%) | 16/313 (5.1%) | 2/313 (0.6%) |
| Sweden | 238 | 225/238 (94.5%) | 7/238 (2.9%) | 6/238 (2.5%) | 227/238 (95.4%) | 5/238 (2.1%) | 6/238 (2.5%) |
| Austria | 211 | 210/211 (99.5%) | 1/211 (0.5%) | 0/211 (0%) | 210/211 (99.5%) | 1/211 (0.5%) | 0/211 (0%) |
| Poland | 188 | 188/188 (100%) | 0/188 (0%) | 0/188 (0%) | 188/188 (100%) | 0/188 (0%) | 0/188 (0%) |
| India | 179 | 159/179 (88.8%) | 13/179 (7.3%) | 7/179 (3.9%) | 161/179 (89.9%) | 10/179 (5.6%) | 8/179 (4.5%) |
| Denmark | 164 | 159/164 (97%) | 4/164 (2.4%) | 1/164 (0.6%) | 151/164 (92.1%) | 12/164 (7.3%) | 1/164 (0.6%) |
| Russian Federation | 154 | 151/154 (98.1%) | 2/154 (1.3%) | 1/154 (0.6%) | 151/154 (98.1%) | 3/154 (1.9%) | 0/154 (0%) |
| Greece | 142 | 140/142 (98.6%) | 0/142 (0%) | 2/142 (1.4%) | 141/142 (99.3%) | 0/142 (0%) | 1/142 (0.7%) |
| Brazil | 125 | 122/125 (97.6%) | 2/125 (1.6%) | 1/125 (0.8%) | 122/125 (97.6%) | 2/125 (1.6%) | 1/125 (0.8%) |
| Israel | 109 | 103/109 (94.5%) | 6/109 (5.5%) | 0/109 (0%) | 101/109 (92.7%) | 8/109 (7.3%) | 0/109 (0%) |
| Norway | 98 | 93/98 (94.9%) | 2/98 (2%) | 3/98 (3.1%) | 95/98 (96.9%) | 2/98 (2%) | 1/98 (1%) |
| Finland | 90 | 89/90 (98.9%) | 1/90 (1.1%) | 0/90 (0%) | 87/90 (96.7%) | 3/90 (3.3%) | 0/90 (0%) |
| Taiwan | 84 | 38/84 (45.2%) | 11/84 (13.1%) | 35/84 (41.7%) | 42/84 (50%) | 8/84 (9.5%) | 34/84 (40.5%) |
| **Country** | **Total, n** | **Correct, n (%)** | **Incorrect, n (%)** | **No Predictions, n (%)** | **Correct, n (%)** | **Incorrect, n (%)** | **No Predictions, n (%)** |
| Czech Republic | 77 | 77/77 (100%) | 0/77 (0%) | 0/77 (0%) | 77/77 (100%) | 0/77 (0%) | 0/77 (0%) |
| Argentina | 76 | 76/76 (100%) | 0/76 (0%) | 0/76 (0%) | 76/76 (100%) | 0/76 (0%) | 0/76 (0%) |
| Hungary | 69 | 69/69 (100%) | 0/69 (0%) | 0/69 (0%) | 69/69 (100%) | 0/69 (0%) | 0/69 (0%) |
| Singapore | 63 | 48/63 (76.2%) | 1/63 (1.6%) | 14/63 (22.2%) | 49/63 (77.8%) | 0/63 (0%) | 14/63 (22.2%) |
| Mexico | 51 | 51/51 (100%) | 0/51 (0%) | 0/51 (0%) | 51/51 (100%) | 0/51 (0%) | 0/51 (0%) |
| Turkey | 47 | 46/47 (97.9%) | 1/47 (2.1%) | 0/47 (0%) | 46/47 (97.9%) | 1/47 (2.1%) | 0/47 (0%) |
| Ukraine | 47 | 43/47 (91.5%) | 3/47 (6.4%) | 1/47 (2.1%) | 44/47 (93.6%) | 3/47 (6.4%) | 0/47 (0%) |
| New Zealand | 43 | 41/43 (95.3%) | 1/43 (2.3%) | 1/43 (2.3%) | 41/43 (95.3%) | 1/43 (2.3%) | 1/43 (2.3%) |
| Ireland | 40 | 40/40 (100%) | 0/40 (0%) | 0/40 (0%) | 40/40 (100%) | 0/40 (0%) | 0/40 (0%) |
| Portugal | 39 | 39/39 (100%) | 0/39 (0%) | 0/39 (0%) | 39/39 (100%) | 0/39 (0%) | 0/39 (0%) |
| Thailand | 29 | 17/29 (58.6%) | 3/29 (10.3%) | 9/29 (31%) | 22/29 (75.9%) | 2/29 (6.9%) | 5/29 (17.2%) |
| Romania | 26 | 26/26 (100%) | 0/26 (0%) | 0/26 (0%) | 26/26 (100%) | 0/26 (0%) | 0/26 (0%) |
| South Africa | 26 | 25/26 (96.2%) | 1/26 (3.8%) | 0/26 (0%) | 24/26 (92.3%) | 2/26 (7.7%) | 0/26 (0%) |
| Chile | 25 | 25/25 (100%) | 0/25 (0%) | 0/25 (0%) | 25/25 (100%) | 0/25 (0%) | 0/25 (0%) |
| Cuba | 22 | 21/22 (95.5%) | 1/22 (4.5%) | 0/22 (0%) | 21/22 (95.5%) | 1/22 (4.5%) | 0/22 (0%) |
| Slovakia | 21 | 20/21 (95.2%) | 0/21 (0%) | 1/21 (4.8%) | 21/21 (100%) | 0/21 (0%) | 0/21 (0%) |
