# Supplementary material for: Inferring gender from first names: Comparing the accuracy of Genderize, Gender API, and the gender R package on authors of diverse nationality
Source: PLOS Digit Health. 2024 Oct 29;3(10):e0000456. doi: 10.1371/journal.pdig.0000456 (PMC11521266; doi:10.1371/journal.pdig.0000456)
Supplement: S10 Table — (DOCX) [file pdig.0000456.s011.docx]

**Supplemental Table 10.** Gender Prediction Accuracy For all Countries with at Least 20 Trialists When Countries are Included in the API Call

|  |  | **Genderize** | | | **Gender API** | | |
| --- | --- | --- | --- | --- | --- | --- | --- |
| **Country** | **Total, n** | **Correct, n (%)** | **Incorrect, n (%)** | **No Predictions, n (%)** | **Correct, n (%)** | **Incorrect, n (%)** | **No Predictions, n (%)** |
| Total | 24929 | 23780/24929 (95.4%) | 520/24929 (2.1%) | 629/24929 (2.5%) | 23922/24929 (96%) | 672/24929 (2.7%) | 335/24929 (1.3%) |
| USA | 9485 | 9049/9485 (95.4%) | 209/9485 (2.2%) | 227/9485 (2.4%) | 9061/9485 (95.5%) | 326/9485 (3.4%) | 98/9485 (1%) |
| France | 1868 | 1810/1868 (96.9%) | 35/1868 (1.9%) | 23/1868 (1.2%) | 1802/1868 (96.5%) | 49/1868 (2.6%) | 17/1868 (0.9%) |
| Germany | 1853 | 1823/1853 (98.4%) | 14/1853 (0.8%) | 16/1853 (0.9%) | 1830/1853 (98.8%) | 14/1853 (0.8%) | 9/1853 (0.5%) |
| Italy | 1754 | 1737/1754 (99%) | 13/1754 (0.7%) | 4/1754 (0.2%) | 1737/1754 (99%) | 14/1754 (0.8%) | 3/1754 (0.2%) |
| United Kingdom | 1713 | 1656/1713 (96.7%) | 19/1713 (1.1%) | 38/1713 (2.2%) | 1686/1713 (98.4%) | 20/1713 (1.2%) | 7/1713 (0.4%) |
| Japan | 1169 | 1146/1169 (98%) | 8/1169 (0.7%) | 15/1169 (1.3%) | 1143/1169 (97.8%) | 10/1169 (0.9%) | 16/1169 (1.4%) |
| Canada | 773 | 737/773 (95.3%) | 12/773 (1.6%) | 24/773 (3.1%) | 756/773 (97.8%) | 10/773 (1.3%) | 7/773 (0.9%) |
| Spain | 726 | 712/726 (98.1%) | 14/726 (1.9%) | 0/726 (0%) | 714/726 (98.3%) | 12/726 (1.7%) | 0/726 (0%) |
| Netherlands | 663 | 639/663 (96.4%) | 13/663 (2%) | 11/663 (1.7%) | 641/663 (96.7%) | 14/663 (2.1%) | 8/663 (1.2%) |
| China | 631 | 482/631 (76.4%) | 93/631 (14.7%) | 56/631 (8.9%) | 487/631 (77.2%) | 110/631 (17.4%) | 34/631 (5.4%) |
| Switzerland | 484 | 457/484 (94.4%) | 8/484 (1.7%) | 19/484 (3.9%) | 470/484 (97.1%) | 6/484 (1.2%) | 8/484 (1.7%) |
| Australia | 475 | 459/475 (96.6%) | 5/475 (1.1%) | 11/475 (2.3%) | 461/475 (97.1%) | 9/475 (1.9%) | 5/475 (1.1%) |
| South Korea | 324 | 256/324 (79%) | 22/324 (6.8%) | 46/324 (14.2%) | 260/324 (80.2%) | 20/324 (6.2%) | 44/324 (13.6%) |
| Belgium | 313 | 294/313 (93.9%) | 9/313 (2.9%) | 10/313 (3.2%) | 299/313 (95.5%) | 12/313 (3.8%) | 2/313 (0.6%) |
| Sweden | 238 | 228/238 (95.8%) | 3/238 (1.3%) | 7/238 (2.9%) | 229/238 (96.2%) | 3/238 (1.3%) | 6/238 (2.5%) |
| Austria | 211 | 209/211 (99.1%) | 2/211 (0.9%) | 0/211 (0%) | 211/211 (100%) | 0/211 (0%) | 0/211 (0%) |
| Poland | 188 | 188/188 (100%) | 0/188 (0%) | 0/188 (0%) | 188/188 (100%) | 0/188 (0%) | 0/188 (0%) |
| India | 179 | 159/179 (88.8%) | 12/179 (6.7%) | 8/179 (4.5%) | 164/179 (91.6%) | 8/179 (4.5%) | 7/179 (3.9%) |
| Denmark | 164 | 159/164 (97%) | 3/164 (1.8%) | 2/164 (1.2%) | 157/164 (95.7%) | 6/164 (3.7%) | 1/164 (0.6%) |
| Russian Federation | 154 | 150/154 (97.4%) | 2/154 (1.3%) | 2/154 (1.3%) | 152/154 (98.7%) | 1/154 (0.6%) | 1/154 (0.6%) |
| Greece | 142 | 138/142 (97.2%) | 0/142 (0%) | 4/142 (2.8%) | 141/142 (99.3%) | 0/142 (0%) | 1/142 (0.7%) |
| Brazil | 125 | 122/125 (97.6%) | 1/125 (0.8%) | 2/125 (1.6%) | 122/125 (97.6%) | 2/125 (1.6%) | 1/125 (0.8%) |
| Israel | 109 | 105/109 (96.3%) | 2/109 (1.8%) | 2/109 (1.8%) | 107/109 (98.2%) | 2/109 (1.8%) | 0/109 (0%) |
| Norway | 98 | 93/98 (94.9%) | 1/98 (1%) | 4/98 (4.1%) | 96/98 (98%) | 1/98 (1%) | 1/98 (1%) |
| Finland | 90 | 86/90 (95.6%) | 2/90 (2.2%) | 2/90 (2.2%) | 85/90 (94.4%) | 5/90 (5.6%) | 0/90 (0%) |
| Taiwan | 84 | 29/84 (34.5%) | 4/84 (4.8%) | 51/84 (60.7%) | 43/84 (51.2%) | 7/84 (8.3%) | 34/84 (40.5%) |
| **Country** | **Total, n** | **Correct, n (%)** | **Incorrect, n (%)** | **No Predictions, n (%)** | **Correct, n (%)** | **Incorrect, n (%)** | **No Predictions, n (%)** |
| Czech Republic | 77 | 77/77 (100%) | 0/77 (0%) | 0/77 (0%) | 77/77 (100%) | 0/77 (0%) | 0/77 (0%) |
| Argentina | 76 | 75/76 (98.7%) | 0/76 (0%) | 1/76 (1.3%) | 76/76 (100%) | 0/76 (0%) | 0/76 (0%) |
| Hungary | 69 | 68/69 (98.6%) | 0/69 (0%) | 1/69 (1.4%) | 69/69 (100%) | 0/69 (0%) | 0/69 (0%) |
| Singapore | 63 | 45/63 (71.4%) | 1/63 (1.6%) | 17/63 (27%) | 49/63 (77.8%) | 0/63 (0%) | 14/63 (22.2%) |
| Mexico | 51 | 51/51 (100%) | 0/51 (0%) | 0/51 (0%) | 51/51 (100%) | 0/51 (0%) | 0/51 (0%) |
| Turkey | 47 | 45/47 (95.7%) | 2/47 (4.3%) | 0/47 (0%) | 46/47 (97.9%) | 1/47 (2.1%) | 0/47 (0%) |
| Ukraine | 47 | 43/47 (91.5%) | 2/47 (4.3%) | 2/47 (4.3%) | 44/47 (93.6%) | 3/47 (6.4%) | 0/47 (0%) |
| New Zealand | 43 | 39/43 (90.7%) | 1/43 (2.3%) | 3/43 (7%) | 40/43 (93%) | 0/43 (0%) | 3/43 (7%) |
| Ireland | 40 | 40/40 (100%) | 0/40 (0%) | 0/40 (0%) | 40/40 (100%) | 0/40 (0%) | 0/40 (0%) |
| Portugal | 39 | 39/39 (100%) | 0/39 (0%) | 0/39 (0%) | 39/39 (100%) | 0/39 (0%) | 0/39 (0%) |
| Thailand | 29 | 17/29 (58.6%) | 2/29 (6.9%) | 10/29 (34.5%) | 22/29 (75.9%) | 2/29 (6.9%) | 5/29 (17.2%) |
| Romania | 26 | 25/26 (96.2%) | 0/26 (0%) | 1/26 (3.8%) | 26/26 (100%) | 0/26 (0%) | 0/26 (0%) |
| South Africa | 26 | 25/26 (96.2%) | 1/26 (3.8%) | 0/26 (0%) | 25/26 (96.2%) | 1/26 (3.8%) | 0/26 (0%) |
| Chile | 25 | 25/25 (100%) | 0/25 (0%) | 0/25 (0%) | 25/25 (100%) | 0/25 (0%) | 0/25 (0%) |
| Cuba | 22 | 21/22 (95.5%) | 1/22 (4.5%) | 0/22 (0%) | 22/22 (100%) | 0/22 (0%) | 0/22 (0%) |
| Slovakia | 21 | 19/21 (90.5%) | 0/21 (0%) | 2/21 (9.5%) | 21/21 (100%) | 0/21 (0%) | 0/21 (0%) |
