# Supplementary material for: Inferring gender from first names: Comparing the accuracy of Genderize, Gender API, and the gender R package on authors of diverse nationality
Source: PLOS Digit Health. 2024 Oct 29;3(10):e0000456. doi: 10.1371/journal.pdig.0000456 (PMC11521266; doi:10.1371/journal.pdig.0000456)
Supplement: Attachment — Submitted filename: point-by-point response R1.docx [file pdig.0000456.s012.docx]

We would like to thank the reviewers for their insightful comments. We have made adjustments accordingly which we believe improve the quality of the manuscript. In the following point-by-point response, we provide each comment from each reviewer in a table, with our response in blue font underneath. We verify that our revised manuscript is not under consideration elsewhere and that all authors agree to the changes. We look forward to continuing to work with you.

**Reviewer 1**

Just kindly re-check once the line 280-283 of Pg-15 as it develops confusion regarding the observed result. Kindly clarify once.

Thanks for catching our mistake. We wrote “Gender API” when we really meant “Genderize.” Accordingly, we changed the sentence:

“Genderize was slightly more accurate than Gender API when countries were not included in the API call. Conversely, Gender API performed slightly better than Gender API when countries were included”

To

“Genderize was slightly more accurate than Gender API when countries were not included in the API call. Conversely, Gender API performed slightly better than Genderize when countries were included”

**Reviewer 2:**

Abstract: incomplete sentence „The accuracy of the gender R package was only evaluated without supplying countries of origin since.“

Thanks for catching our mistake. We changed the sentence to:

“The accuracy of the gender R package was only evaluated without supplying countries of origin.”

Line 55: Why are gender gaps in the authorship (not patient inclusion) of cancer clinical trials of particular concern?

Thank you for the question. Gender gaps in clinical trial patient participation is an important factor that should be studied. However, gender ratios of clinical trial participants are outside of the scope of our study, which focuses on named authors. With extremely few exceptions from the older literature, patient names are not routinely provided.

Lines 65-66, 80-81, 327: How did the authors define Western / Non-Western names? The present approach only evaluated names from Western / Asian countries.

Thank you for your question. In this analysis we used the country of origin as a surrogate for author name origin. As noted in our discussion of limitations, “…a researcher’s nationality in our data set does not always reflect the cultural origin of their first name as some researchers immigrated to the country of their academic affiliation.”

We have modified our category names in the manuscript to improve clarity. We have replaced every instance of “Western” with “non-Asian”, and every instance of “non-Western” with “Asian.”

Lines 106-110: The R package only returns aggregate percentages for each name. The authors should describe in greater detail how they calculated the numbers in Table 1 based on these aggregate percentages. Did they a) multiply probabilities with total number of persons with given name and round the result or b) assume that all persons of a given name belong to the same gender? Example: index = 53717 / first_name = ilya / probability = 0.5008 / predicted_gender = woman / man_count = 2 / woman_count = 0. In this example scenario a) would yield 1 man and 1 woman (accuracy 50%) while scenario b) would yield 0 man and 2 woman (accuracy 0%).

We have added additional sentences to the methods section to clarify:

“The gender R package provides gender probabilities rather than explicit gender predictions. We assigned gender based on which gender was reported to have the highest probability in the R package. For example, the name Mark was classified as a man’s name because the gender R package returned a 99.7% probability that ‘Mark’ is a man when the IPUMS method is selected.

Lines 145-151: The addition of a flow chart depicting inclusion/exclusion of trialists as described would be helpful for the reader.

Thanks for pointing out that our methods section requires greater transparency regarding how trialists are added to the HemOnc knowledgebase. It is important to note that the process of adding trialists to the HemOnc knowledgebase features only inclusions.

To that end, we added the following sentences to our methods section to funnel readers to additional resources if they want to learn more about the creation of the HemOnc knowledgebase:

“The methodology for building the HemOnc knowledgebase has been previously described [16]. Likewise, the edibility criteria for including trialists in the HemOnc knowledgebase is defined on HemOnc.org [17].”

Additionally, we also added this supplemental figure to the results section to visualize how we selected trialists to include in our study:

**Supp. Fig 1: Flowchart depicting trialist inclusions from HemOnc Knowledgebase (KB).** The HemOnc KB is a growing resource, and 4,360 trialists had not had their genders evaluated at the time of this study. Of the 32,968 trialists included in the study, 24,930 were affiliated with sites in a single country.

Lines 171-173: A supplementary list of the most common names that yielded incorrect predictions would be useful for later studies.

Excellent suggestion. We added supplementary tables that include all names that were misgendered more than once. To be more specific, we added tables for the following methods:

- Genderize without country
- Genderize with country
- Gender API without country
- Gender API with country
- gender R package SSA method
- gender R package IPUMS method

Lines 174-175 misses a direct statistical comparison between all methods used. Only comparisons between Genderize vs. Gender API and SSA vs. IPUMS are reported.

We compared the accuracy of the gender R package to the proprietary methods and added the following sentence to the results:

“The accuracy differences between the gender R package methods and both Genderize and Gender API were significant, in favor of Genderize and Gender API in all cases (p<0.001).”

Lines 176-177: For the sake of clarity, these lines should be moved to the result section that compares the different delimiters.

We paraphrased lines 176-177 at the beginning of the section that compares delimiters:

“24,930 of the 32,968 (75.6%) trialists in our dataset were affiliated with sites in a single country and were assigned to the country of their affiliation when querying Genderize and Gender API with nationalities.”

Line 207: Figure 1 should include bar graphs and statistical comparisons for the R package options that were investigated

In response to your suggestion, we initially tried adding a third panel to figure 1 showing the accuracy of the gender R package. However, we found that this strategy made the figure too large. We opted to add another figure following figure 1 that shows the accuracy of the gender R package:

**Fig 2: Accuracy of the gender R Package.** Columns a and b show the accuracy of the gender R package’s SSA and IPUMS methods respectively. The top 4 countries with the most trialists and Singapore, Japan, China, Taiwan, and South Korea are plotted. Only trialists affiliated with sites in a single country are plotted.

Line 274: Please provide year and month of this cost analysis as prices can be highly dynamic.

We added the year and month:

“The costs mentioned herein are accurate as of April 2024.”

What year or range of years did the authors impute into the R algorithm? This could greatly impact the accuracy of the algorithm.

We added the following section to the methods section to clarify:

“The SSA and IPUMS methods source names from 1932 to 2012 and 1789 to 1930 respectively [20].”

**Reviewer 3:**

Why and when names-based gender identification needs to be used, including the limitations and potential biases inherent in this approach

We added a section to the discussion that mentions use cases for gender inference tools:

“Tools [22,23] that infer gender from first names are commonly used by meta researchers to evaluate gender ratios in academia. A study [31] by Holman et al in 2018 reported persistent gender disparities in traditionally male-dominated fields. Gender inference technology like Genderize and Gender API facilitate programmatic gender mapping for methodologies that evaluate gender ratios based on first names.”

Additionally, we elaborated on the limitations and biases in our discussion of how non-binary people are excluded by methodologies that use the gender inference tools we evaluated.

“Creating tools that predict the likelihood that an individual is non-binary based on first names is challenging, due in part to the lack of gender labeled datasets that include non-binary individuals. We suggest that academic journals could facilitate research on gender equity among clinical trialists and academia at large by including self-reported author gender identities. Critically, methodologies that use tools that infer binary gender must acknowledge the exclusion of non-binary individuals as a limitation. Furthermore, we recommend that researchers prioritize accuracy over cost when selecting gender inference tools in order to reduce the number of names that are misgendered.”

An in-depth analysis of the methodological challenges, particularly concerning names that are gender-neutral, and an estimation of the maximum accuracy achievable under these conditions.

We added a section to the discussion that mentions how gender inference tools will never be 100% accurate due to gender-neutral names:

“Tools that infer gender from first names will never achieve perfect accuracy because many names are gender-neutral. additionally, the gender of certain names correlates with country of origin. For example, Andrea, Gabriele, and Daniele are more likely to correspond to men if they are of Italian origin. In fact, Andrea was the most frequently misgendered name when Genderize and Gender API were called without including countries of origin.”

Unfortunately, we are not able to quantify the unavoidable inaccuracies in gender inference caused by gender-neutral names. This is because we do not have any method to differentiate inaccuracies due to problems with gender inference tools from inaccuracies that stem from gender-neutral names.

Identifying names that are frequently misclassified would enhance the paper's insightfulness.

Excellent suggestion. We added supplementary tables that include all names that were misgendered more than once. To be more specific, we added tables for the following methods:

- Genderize without country
- Genderize with country
- Gender API without country
- Gender API with country
- gender R package SSA method
- gender R package IPUMS method

Recommendations for more effective methods, such as integrating / combining multiple approaches or adding more contextual information to enhance prediction accuracy, should be explored.

You provide a very interesting idea. An end user could hypothetically evaluate a dataset of names first with the gender R package. Then, names that were not classified by the gender R package could be evaluated with Genderize or Gender API. This strategy would be cheaper than using only Genderize or Gender API because the gender R package is free. However, we would not recommend this strategy in practice because sacrificing accuracy for cost is probably not a good trade off since Genderize and Gender API are fairly cheap to begin with.

Regarding your suggestion that we could enhance accuracy by adding more contextual information, unfortunately this is not possible with Genderize or Gender API beyond supplying countries of origin to the API.

The impact of including the country of origin on prediction accuracy needs further examination, including whether specific names significantly affect the results.

Great suggestion. We added the following section to the results in order to further elucidate the effect of including countries in the API call:

“All names that were misgendered more than once are catalogued in supplementary tables 1 through 6. The name Andrea was most frequently misgendered when calling the Genderize and Gender API services without countries in the API call. Jan was the most misgendered name for the gender R package SSA method and Genderize when countries were included. The most frequently misgendered names for Gender API when countries are included and the gender R package IPUMS method were Laurence and Nicole respectively.”

Additionally, we analyzed the implications of these results in the following discussion section:

“Tools that infer gender from first names will never achieve perfect accuracy because many names are gender-neutral. additionally, the gender of certain names correlates with country of origin. For example, Andrea, Gabriele, and Daniele are more likely to correspond to men if they are of Italian origin. In fact, Andrea was the most frequently misgendered name when Genderize and Gender API were called without including countries of origin.”

The discussion on non-binary gender options requires clarification on how they integrate with name-based gender prediction methods, questioning the assumption that certain names can indicate non-binary identities.

After considering your comment, we have decided that it is impractical to suggest that tools like Genderize and Gender API could be modified to predict non-binary identities. Consequently, we removed the following section from the manuscript:

“Going forward, tools that infer gender based on name should be trained on data that include transgender and nonbinary people, and they should include the option to predict an individual as non-binary or transgender. Gender prediction is not simply a binary classification problem. Transgender individuals will likely make up a small percentage of the dataset of names, which could make obtaining a correct prediction for these individuals very challenging. Yet, by not incorporating them, we are excluding countless people from this algorithm and ensuring the prediction of their gender to have a 0% accuracy.”

This topic should extend into a broader dialogue on methodological limitations, citing examples like Meryll, Parker, and Jamie, which are ambiguous in terms of gender.

We added a section to the discussion that mentions the implications of gender-neutral names:

“Tools that infer gender from first names will never achieve perfect accuracy because many names are gender-neutral. Additionly, the gender of certain names correlates with country of origin. For example, Andrea, Gabriele, and Daniele are more likely to correspond to men if they are of Italian origin. In fact, Andrea was the most frequently misgendered name when Genderize and Gender API were called without including countries of origin.”

Suggestions for improving accuracy, particularly in regions where current methods show diminished effectiveness, would greatly contribute to the paper's value.

Our only suggestion for improving accuracy is to manually evaluate gender predictions that Genderize or Gender API deemed had a low probability of being accurate. Likewise, end users could also manually evaluate names that yielded no prediction by gender inference services. To this end, we suggested in the manuscript that all scientists should have a public presence that could be used to manually infer their gender:

“The ambiguity of gender-neutral names underscores the importance for researchers to have a public presence that includes self-reported demographic information.”

Exploring how the accuracy of the model might shift over time, in response to changes in the popularity of names, would also be insightful.

After some thought, we believe that changes in the accuracy of gender inference models over time will far outweigh trends in the popularity of gender-neutral names. We discussed this thought in the following sentence:

“Importantly, the higher accuracy achieved after reanalyzing Sebo and Santamaria’s datasets suggests that the accuracy of Genderize and Gender API is likely to increase over time as their respective companies continue to improve their products. Indeed, algorithms such as Genderize and Gender API must be periodically calibrated in order to maintain their accuracy in changing contexts [32,33].”

**Reviewer 4**

Although the manuscript follows the IMRaD format (Introduction, Method, Results and Discussion), which is a widely recognized structure used in scientific writing, especially in journals such as PLOS ONE, I missed an independent and robust literature review section.

Great suggestion. We added a brief literature review section to the discussion:

“The present study provides an updated accuracy benchmark for tools that infer gender from first names. Furthermore, this study adds to a growing body of literature on the accuracy of gender inference tools. Santamaria and Mihaljevic compared Gender API, gender-guesser, Genderize, NameAPI, and NamSor in 2018, and concluded that Gender API achieved superior accuracy [15]. In 2021, Sebo compared Gender API, Genderize, and NamSor, ultimately reporting that Gender API demonstrated the highest accuracy, followed by NamSor [21].”

The authors rely on a dataset of clinical trial authors sourced from the HemOnc knowledge base, which may not accurately represent the diversity of names and genders worldwide [I am not sure]. Can you please elaborate on this subject?

Great question. Our dataset reflects the diversity of clinical trial authors but does not reflect gender diversity worldwide. We added the following sentence to clarify:

“Furthermore, the database of gendered researchers in the HemOnc KB is constrained by the diversity of the clinical trialist community.”

The article needs to address the limitations of binary gender classification and the exclusion of transgender and nonbinary individuals from the analysis. This reflects a broader issue of gender essentialism. Thus, the article treats gender as a binary variable without critically engaging with the social construction of gender and its fluidity. By reinforcing binary gender norms, the article perpetuates outdated and harmful stereotypes, neglecting the diverse experiences and identities of individuals across the gender spectrum.

Excellent point. We modified the section of our manuscript that discusses non-binary individuals to better advocate for their inclusion:

“It is important to note that Genderize, Gender API, and the gender R package assume a gender binary. However, a recent survey [37] found that 1.6% of U.S. adults identify as transgender or nonbinary. With new algorithmic advancements such as Genderize and Gender API, it is imperative that inclusivity is incorporated. Creating tools that predict the likelihood that an individual is non-binary based on first names is challenging, due in part to the lack of gender labeled datasets that include non-binary individuals. We suggest that academic journals could facilitate research on gender equity among clinical trialists and academia at large by including self-reported author gender identities. Critically, methodologies that use tools that infer binary gender must acknowledge the exclusion of non-binary individuals as a limitation. Furthermore, researchers should consider prioritizing accuracy over cost when selecting gender inference tools in order to reduce the number of names that are misgendered.”

The comparison between the accuracy of Genderize and Gender API appears to ignore critical specificities regarding names from non-Western cultures. While the study acknowledges the lower accuracy of these tools with Asian names, it does not provide meaningful information about the reasons behind this disparity or propose solutions to resolve it.

We added an additional section to the manuscript that discusses possible reasons why Asian names were more often misgendered. Additionally, we suggest ways in which journals and researchers could make their gender identity more transparent for equity researchers. Here is the new passage:

“All tools we evaluated were least accurate with names from Asian countries, excluding Japan. Many Asian names with an identifiable gender in their native script become a gender-neutral representation when converted to the Latin alphabet [15]. Journals should consider publishing Asian names using native characters in addition to English translations. The ambiguity of gender-neutral names underscores the importance for researchers to have a public presence that includes self-reported demographic information. ”

Furthermore, the article's emphasis on cost-effectiveness ignores ethical considerations and the potential consequences of prioritizing accessibility over accuracy in gender prediction.

We added the following sentence to the discussion to clarify our position on prioritizing accuracy of cost:

“Furthermore, researchers should consider prioritizing accuracy over cost when selecting gender inference tools in order to reduce the number of names that are misgendered.”

The article could benefit from a discussion of future research directions and potential improvements in gender prediction methodologies. It could include exploring alternative approaches such as probabilistic modeling, incorporating user feedback and community input, and integrating intersectional perspectives to address the limitations of existing tools. In essence, I believe that the article has the potential to significantly enhance its robustness in its present form. Any limitations that cannot be adequately addressed at this stage should be acknowledged, and avenues for further research should be suggested for future exploration.

This is a very interesting suggestion. However, it is challenging to suggest ways to improve Genderize and Gender API because how these tools work are trade secrets. At present, we do not believe that we have enough information to offer recommendations to the companies that develop Genderize and Gender API. Furthermore, it is challenging to suggest future development directions for Genderize and Gender API because we do not know how the current versions work.

**Reviewer 5:**

The concluding statement of the abstract needs rephrasing to sum up the findings.

Great suggestion. We modified the concluding sentence of the abstract in order to sum up our results:

“Our results indicate that Genderize and Gender API achieve similar accuracy on a multinational dataset. The gender R package is uniformly less accurate than Genderize and Gender API.”

In the methods: The list of definitions could be placed as a separate section

Thanks for your comment. We added subsection headers to the methods section, including a subsection with definitions called “Evaluation metrics.”

Make sure all the criteria are defined in the method for e.g., high profile journals

We added two sentences to the methods section that provide resources for readers that want to learn more about the process for building the HemOnc knowledgebase:

“The methodology for building the HemOnc knowledgebase has been previously described [16]. Likewise, the edibility criteria for including trialists in the HemOnc knowledgebase is defined on HemOnc.org [17].”

Discuss in detail the differences in predictions between the different tools used. This is shown in the results communicated but not discussed thoroughly

Thanks for the suggestion. Upon revisiting the discussion, we realized that we discussed the results of Genderize and Gender API but omitted results pertaining to the gender R package. We added a paragraph to the discussion section to fill in this gap:

“The gender R package was less accurate than both Genderize and Gender API on all fronts (Table 1, Fig 1, Fig 2). Between the two gender R package methods, the SSA method proved more accurate. Interestingly, the SSA method misgendered men as women more frequently than the reverse. In contrast, all other tools evaluated in this study misgendered women as men more frequently.”

In the discussion, highlight the performance with non-Western names

Thanks for the suggestion. We added a section to the discussion that highlights the lower performance with Asian names:

“All tools we evaluated were least accurate with names from Asian countries, excluding Japan. Many Asian names with an identifiable gender in their native script become a gender-neutral representation when converted to the Latin alphabet [15]. Journals should consider publishing Asian names using native characters in addition to English translations. The ambiguity of gender-neutral names underscores the importance for researchers to have a public presence that includes self-reported demographic information.”

**Reviewer 6:**

How does api's work?

Unfortunately, how Genderize and Gender API work under the hood are trade secrets.

what is the basis for comparing these apis?

We added a paragraph to the discussion that clarifies the rationale for comparing the accuracy to these gender inference tools:

“Tools [22,23] that infer gender from first names are commonly used by meta researchers to evaluate gender ratios in academia. A study [31] by Holman et al in 2018 reported persistent gender disparities in traditionally male-dominated fields. Gender inference technology like Genderize and Gender API facilitate programmatic gender mapping for methodologies that evaluate gender ratios based on first names.”

How does the internal structures of these apis influence these comparisons?

It is challenging to speculate how the internal structure of these APIs influence our comparisons because how the APIs work are trade secrets.

Concerning the choice of these api's, what are their related differences?

Thanks for your question. We discussed some of the differences between these tools in the “Cost and Accessibility” section. It is challenging to comment further on the differences between these APIs than we did because the inner workings of Genderize and Gender API are trade secrets.

what are the study constraints and identified bias.

Great question. We discussed all the limitations we could think of in the limitations section. additionally, we elaborated on how Genderize and Gender API exclude non-binary individuals in the following section:

“Creating tools that predict the likelihood that an individual is non-binary based on first names is challenging, due in part to the lack of gender labeled datasets that include non-binary individuals. We suggest that academic journals could facilitate research on gender equity among clinical trialists and academia at large by including self-reported author gender identities. Critically, methodologies that use tools that infer binary gender must acknowledge the exclusion of non-binary individuals as a limitation. Furthermore, researchers should consider prioritizing accuracy over cost when selecting gender inference tools in order to reduce the number of names that are misgendered.”

**Reviewer 7:**

It is unclear what is the significance or relevance to digital health. For example, is this helping to improve the accuracy of trials?

Excellent question. We believe that it is important for clinical trial authorship teams to include individuals with diverse gender identities. Since cancer impacts everyone regardless of gender, it is important that many voices play a role in interpreting the results of clinical trials.

How could this improve the recognition of gender through first names? Evaluating packages/tools may carry practical value, but it needs to be highlighted

Great question. We added a sentence to the discussion that mentions some use cases for these tools:

“Tools [22,23] that infer gender from first names are commonly used by meta researchers to evaluate gender ratios in academia. A study [31] by Holman et al in 2018 reported persistent gender disparities in traditionally male-dominated fields. Gender inference technology like Genderize and Gender API facilitate programmatic gender mapping for methodologies that evaluate gender ratios based on first names.”

Also, the statistical method used is very basic.

Our primary goal for including statistical tests was to demonstrate that the differences we measured between gender inference methods would not disappear if we increased our sample size of clinical trialists. We believe that the tests we used are sufficient for that purpose.
