# Supplementary material for: Inferring gender from first names: Comparing the accuracy of Genderize, Gender API, and the gender R package on authors of diverse nationality
Source: PLOS Digit Health. 2024 Oct 29;3(10):e0000456. doi: 10.1371/journal.pdig.0000456 (PMC11521266; doi:10.1371/journal.pdig.0000456)
Supplement: Attachment — Submitted filename: PLOS Digital Health Letter R2.docx [file pdig.0000456.s013.docx]

RE: **Revisions for PDIG-D-24-00041R1 - "Inferring Gender from First Names: Comparing the Accuracy of Genderize, Gender API, and the gender R Package on Authors of Diverse Nationality"**

Thank you for the opportunity to revise and resubmit this manuscript. We are extremely appreciative of the time and effort that the reviewers and editorial team have devoted to our manuscript, and believe the feedback provided has substantially strengthened our submission. Please find a point-by-point response (reviewers’ comments in black, response in blue; line numbers correspond to the tracked changes document) to the issues raised by the reviewers and editorial team below. Additionally, we made some adjustments to supplemental figure 1 to improve clarity. In the previous version of supplemental figure 1, some of the trialist exclusions were mentioned in the figure caption, but not included in the flowchart. We added those exclusions to the flowchart.

**Reviewer #8:**

COMMENTS NOT ADDRESSED FULL

- Specific comments appear to have been redacted. We are unclear which of the comments have not yet been addressed to satisfaction. We will appreciate additional information, if applicable.

**Reviewer #10:**

Accuracy alone can be misleading, especially in datasets with class imbalance. Precision, recall, and F1 score provide a more detailed understanding of the performance by considering the trade-offs between false positives and false negatives.

- Excellent suggestion. We added this paragraph to the methods section:

Lines 144-148:

“Precision, recall, and both gender-specific and global F1 scores were calculated for each gender inference tool. For gender-specific F1 scores, one gender was designated the positive prediction, and the other gender the negative prediction. Gender-specific F1 scores were calculated for both men and women. For global F1 scores, correctly gendered Men’s names and correctly gendered women’s names were classified as true positives. Names that yielded no gender predictions were classified as false negatives.”

Additionally, we added this sentence to the results section:

Lines 206-207:

“Precision, Recall, and F1 scores for all gender inference tools are presented in **Supplementary Table 8**.”

The authors have noted the presence of missing values present in the data, Trialists with unresolved names or undetermined genders were excluded from the analysis. The authors should provide a detailed explanation of how this exclusion might affect the results. The exclusion of a significant proportion of trialists with missing data (7.1% unresolved names, 11.9% unresolved genders) could introduce bias, especially if the missing data is not random.

- Thank you for this feedback. We have now elaborated the relevant section in the discussion which explains how excluded names might introduce bias into our results. This section now mentions that 18.1% of names were without a determined gender. Hopefully, our discussion of bias is now clearer after the changes (lines 335-336).

The introduction should briefly discuss the importance of accurate gender prediction in broader contexts beyond academia, such as implications for public health, policy-making, and diversity metrics.

- Great suggestion. We added the following sentence to our introduction:

Lines 71-74:

“Furthermore, gender disparities in healthcare accessibility have also been documented, and gender disparity also affects public health, policy-making, and diversity metrics.”

The manuscript should provide a more in-depth analysis of why non-Western names, especially from Asian countries, are less accurately predicted. This should include a discussion of cultural and linguistic factors affecting name-gender associations.

- Thanks for the suggestion. We have bolstered our discussion of the cultural and linguistic factors that explain the lower prediction accuracy of Asian names with the following sentences:

Lines 348-351

“Even so, a 2022 study found that many Chinese characters that are traditionally used in women’s names have become more gender-neutral over time [ref]. Similarly, a 2023 study reported that phonological characteristics that indicate the gender of the English spelling of Korean names have also become more ambiguous post-2020 [ref].”

Expand the discussion on the limitations of using binary gender classifications and the exclusion of non-binary and transgender identities. This section should also discuss the potential biases introduced by these limitations and suggest ways future research can address them.

- Thanks for the suggestion. We expanded our discussion of non-binary individuals and how to include them in meta-research studies (lines 359-377).

**Reviewer #11:**

I think this paper is well-written and transparent, with an appropriate statistical analysis and that its conclusions follow from the analysis. The figures and tables are all appropriate.

However, I think the paper has a fundamental flaw that makes it of limited usefulness which I will try to explain here. From examination of the Genderize and Gender API websites, as well as the source code of the gender package (and the genderdata package on which it depends), it appears that all three "methods" discussed in the paper are actually the same method: a simple look-up of the given name on the database associated with the method. Of course, there is some simple text-parsing with the API-based tools that allows the input to be less sensitive to formatting (in particular with Gender API), but otherwise this study is comparing the accuracy of four databases, not three methods. The R package actually allows the user to access the Genderize database by specifying method = "genderize" rather than method = "ssa" or method = "ipums" inside the gender() function, and this would presumably give identical results to those reported for Genderize in this paper.

- Thank you for a rigorous and thoughtful review. In our following responses, we hope to convince you that our paper provides value to meta-researchers. Our goal when writing this paper was not to compare the algorithms of gender inference tools. Instead, our goal was to create an accuracy benchmark that meta-researchers can use to inform which gender prediction tool they choose.

To deemphasize the algorithmic aspects of gender inference tools, we have changed the sentence on lines 361-362.

- We agree that Genderize, Gender API, and the Gender R packages most likely predict gender using look up tables under the hood. However, we don’t think the apparently simple design of these tools detracts from the usefulness of our paper. End users of gender inference tools will gravitate to the most accurate tool. Whether that tool’s superior performance is due to a superior algorithm, or a more complete lookup table, is not necessarily important to the target audience of our paper. Furthermore, we are unable to discuss how these gender inference tools work because Genderize and Gender API are not open source, so discussing how they operate under the hood would be speculative.

The R package also contains two other historical databases, namely the North American Population Project (NAPP) database, and the

Kantrowitz name corpus database. Neither are even mentioned in this paper for some reason.

- Thank you for noting this. We added the following section to the discussion:

Lines 354-358:

“In this study, the gender R package was tested with the SSA and IPUMS methods because those name datasets are more recent than the gender R package’s NAPP method, which does not include names post-1910. The gender R package’s Kantrowitz method was also not used because potentially gender-neutral names like Alex, Jamie, and Andrea listed as having a gender of “either”, without a probability of which gender is more likely.”

That being the case, it seems the paper is actually comparing four databases: the actively commercially maintained Gender API database, the actively commercially maintained Genderize database, the historical, unmaintained SSA database and the even older unmaintained IPUMS database. The paper's conclusion, namely that Genderize and Gender API are more accurate than the gender R package, is saying little other than actively maintained and up-to-date name databases are more accurate than unmaintained historical databases that have not been updated for many years. This should surprise no-one, yet that is not how the results are presented.

- While the superior accuracy of Genderize and Gender API compared to the gender R package is unsurprising, we believe it is important to highlight just how substantially more accurate proprietary gender inference tools are compared to their open-source counterpart. It is important for end users to know the magnitude of the accuracy differences between the gender R package and Genderize/Gender API so they can weigh the pros and cons of free open-source tools compared to paid proprietary options. We added the following sentence to the discussion to communicate this rationale to our readers”

Lines 290-292:

“The high accuracy of Genderize and Gender API, coupled with relatively modest usage fees, suggest that these proprietary tools may be superior to the gender R package for many use cases.”

Because the paper treats the APIs like a "black box", the casual reader would be left with the impression that they are something more sophisticated than they actually are, which is simply well-maintained lookup tables. Furthermore, the R package can presumably be induced to equalling their accuracy with a simple change of input parameters and an API key for the Genderize database, so framing this as the R package being less accurate is just misleading. It is the open source data to which the package has free access that is lacking, not the software itself.

- We respectfully disagree with the reviewer on this point. The gender R package provides only limited functionality of the genderize API. For example, the gender R package does not allow the user to call the genderize API with countries of origin. Furthermore, the gender R package does not allow the user to include a genderize API key, which limits the number of API calls a user can perform per day.
- Whether or not the poorer performance of the gender R package is the result of software or underlying data is not within the scope of this study to evaluate. We are reporting quantifiable performance differences between gender inference tools. Additionally, the low accuracy of the gender R package could be exacerbated by name formatting, such as first name-last name combinations. However, API-specific features such as Gender API’s text parsing capabilities can substantially improve performance related to inconsistent name formatting.

In addition, I have concern about the longevity of any conclusions drawn from this study. APIs change, companies go bust, R packages have a fairly high attrition rate too (maybe 40% per 10 years). This paper describes a snapshot of three currently available methods to predict gender from names that are very unlikely to all stay in their current format for more than few years, particularly with the advent of context-rich AI analysis.

On that subject, it is entirely possible that even the current iteration of ChatGPT could give a more accurate gender prediction than any of the methods discussed here, and this will only improve with time. Ultimately, a medical / sociological scientific report on the accuracy of inscrutable proprietry method that is subject to change and cannot be replicated does little to advance our knowledge.

- We agree that the accuracy of gender inference tools evolve over time. As part of our study, we replicated a 2018 study, and found that Genderize and Gender API have improved their accuracy. It is important that updated accuracy benchmarks for gender inference tools are provided to the meta-research community at regular intervals so that researchers can design optimal methodologies. Without updated accuracy benchmarks, meta-researchers could continue to rely on outdated benchmarks. We added the following sentence to the end of the discussion to reinforce for the reader that Genderize and Gender API are improving their usefulness:

Lines 386-389:

“Replicating the studies of Santamaria 2018 and Sebo 2021 demonstrated that Genderize and Gender API have improved over time. Accordingly we should expect that gender prediction accuracy and features of Genderize and Gender API would continue to be more accurate over time.”

- Regarding the ChatGPT suggestions, we thank the reviewer for this interesting idea. However, using ChatGPT or other LLMs to infer gender is outside the scope of this study. We did conduct some informal limited evaluation of commercial LLMs and found that they suggested the wrong gender nearly as often as the correct gender for authors with gender-neutral names. As this evaluation was informal and was not further utilized, we do not mention LLMs in the body of the manuscript.
- Finally, we feel that the determination of whether or not our manuscript advances knowledge is ultimately up to the scientific community. Since it was released in 2024-01-31, our manuscript’s preprint (<https://doi.org/10.1101/2024.01.30.24302027>) has been cited four times. These citations suggest that the scientific community has found value in our analyses.
